# MutaGAN: A sequence-to-sequence GAN framework to predict mutations of evolving protein populations

**DOI:** 10.1093/ve/vead022

**Published:** 2023-04-07

**Authors:** Daniel S Berman, Craig Howser, Thomas Mehoke, Amanda W Ernlund, Jared D Evans

**Affiliations:** Johns Hopkins Applied Physics Laboratory, 11100 Johns Hopkins Rd., Laurel, MD 20723, USA; Johns Hopkins Applied Physics Laboratory, 11100 Johns Hopkins Rd., Laurel, MD 20723, USA; Johns Hopkins Applied Physics Laboratory, 11100 Johns Hopkins Rd., Laurel, MD 20723, USA; Johns Hopkins Applied Physics Laboratory, 11100 Johns Hopkins Rd., Laurel, MD 20723, USA; Johns Hopkins Applied Physics Laboratory, 11100 Johns Hopkins Rd., Laurel, MD 20723, USA

**Keywords:** generative adversarial networks, sequence generation, Influenza virus, deep learning, evolution

## Abstract

The ability to predict the evolution of a pathogen would significantly improve the ability to control, prevent, and treat disease. Machine learning, however, is yet to be used to predict the evolutionary progeny of a virus. To address this gap, we developed a novel machine learning framework, named MutaGAN, using generative adversarial networks with sequence-to-sequence, recurrent neural networks generator to accurately predict genetic mutations and evolution of future biological populations. MutaGAN was trained using a generalized time-reversible phylogenetic model of protein evolution with maximum likelihood tree estimation. MutaGAN was applied to influenza virus sequences because influenza evolves quickly and there is a large amount of publicly available data from the National Center for Biotechnology Information’s Influenza Virus Resource. MutaGAN generated ‘child’ sequences from a given ‘parent’ protein sequence with a median Levenshtein distance of 4.00 amino acids. Additionally, the generator was able to generate sequences that contained at least one known mutation identified within the global influenza virus population for 72.8 per cent of parent sequences. These results demonstrate the power of the MutaGAN framework to aid in pathogen forecasting with implications for broad utility in evolutionary prediction for any protein population.

## Introduction

Biological evolution mainly manifests itself through seemingly random mutations that occur during genome replication. When this change improves organismal fitness, the probability the mutation is passed on to future generations is increased. Virus replication is inherently error-prone, and only mutations that maintain the ability to infect hosts and evade the host immune system are inherited by subsequent generations. Because these mutations occur seemingly randomly in the genetic sequence that codes for these proteins, it is difficult to predict which strains will emerge and become predominant.

Although it is not currently possible to capture all variables that give rise to traits across a population, modeling the appearance and persistence of different mutations over time can serve as a proxy for understanding environmental pressures (Frank and Slatkin 1990; Kussell and Leibler 2005; Wolf, Vazirani, and Arkin 2005; Mustonen and Lässig 2009). Subsequently, if an accurate model can be created, changes that occur in future populations can be predicted (Bedford, Rambaut, and Pascual 2012; Neher, Russell, and Shraiman 2014; Neher et al. 2016). Tools to predict the evolution of a biological organism would significantly improve our ability to prevent and treat disease. The knowledge of how an organism will evolve would allow us to develop more precise interventions and preventive measures in advance and to prevent outbreaks or combat invasive species. Deep learning has led to performance breakthroughs in a number of applications but is yet to contribute to predicting mutations and evolution of biological populations. We viewed this problem of predicting mutations as analogous to some natural language processing (NLP) tasks, like translation and text generation, for which deep learning has proven successful, making it a great model candidate.

### Deep learning and biological sequences

New methods in data science have been applied to biological sequences for purposes of unsupervised characterization and supervised classification tasks. Deep learning is a natural candidate for these efforts due to an exceptional ability to abstract higher-order structures from high-resolution and complex datasets. Previous work has applied NLP techniques to genomic sequence sets (Bengio et al. 2003; Bengio, Courville, and Vincent 2013; Mikolov, Yih, and Zweig 2013; Mikolov et al. 2013a,b; Levy and Goldberg 2014). Ng created a word embeddings process for DNA, called dna2vec, which creates vector representations for short substrings of DNA sequences (Ng 2017). The extension of deep neural network architectures such as convolutional neural networks (CNNs), recurrent neural networks (RNNs), and stacked autoencoders onto biological sequence data has proven useful for DNA sequence classification (Rizzo et al. 2015; Zhou and Troyanskaya 2015; Quang and Xie 2016) as well as prediction of RNA binding sites (Alipanahi et al. 2015), protein–protein interactions (Sun et al. 2017), and DNA–protein binding (Zeng et al. 2016). Furthermore, deep learning methods have been extended to the problem of protein-folding (Spencer, Eickholt, and Cheng 2014; Asgari and Mofrad 2015) to predict molecular characteristics like secondary structure (Wang et al. 2016), backbone angle and solvent accessibility surface areas (Heffernan et al. 2015), and other details about proteins (Bepler and Berger 2019).

In 2014, Goodfellow et al. developed a technique for training generative models called generative adversarial networks (GANs) (Goodfellow et al. 2020), followed by the development of conditional GANs in the same year (Mirza and Osindero 2014). GANs have seen the greatest success in image generation (Reed et al. 2016; Isola et al. 2017; Ledig et al. 2017; Ma et al. 2017) and have also been used to generate text (Yu et al. 2017; Zhang et al. 2018; Keneshloo et al. 2019; Tuan and Lee 2019). GANs have also been extended to bioengineering applications, where they were implemented in conjunction with CNNs to optimize DNA for microarray probe design (Killoran et al. 2017), protein sequences for discovery of novel enzymes (Repecka et al. 2021), as well as implemented with an RNN for gene sequence optimization for antimicrobial peptide production (Gupta and Zou 2018), all from random noise. Additionally, a CNN-based GAN was used to predict the most probable folding of protein sequences given amino acid sequence and pairwise distances between α-carbons on the protein backbone (Anand and Huang 2018). In all of these cases, sequence length ranged between 50 and 300 amino acids. However, none of these used an RNN conditional GAN to model the natural evolution of a biological sequence.

### Sequence-to-sequence model

**Figure 1.**
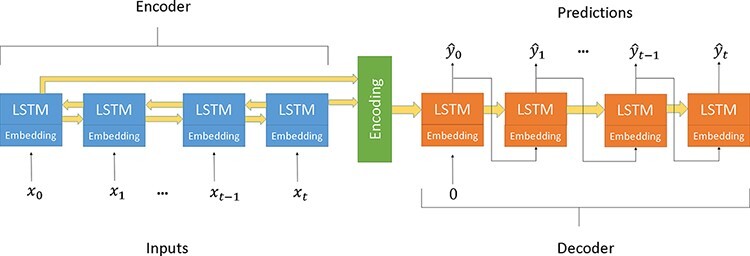
A seq2seq model using two bidirectional LSTM encoder and a unidirectional LSTM decoder and embedding layers.

The specific deep learning architecture used to enable high-performance encoded representations of sequences is known as a sequence-to-sequence (seq2seq) model (Hochreiter and Schmidhuber 1997). A seq2seq model is a type of neural machine translation algorithm that uses at least two RNNs, like long short-term memory (LSTMs) (Sutskever, Vinyals, and Le 2014), that take as input a sequence with the goal of constructing a new sequence (Sutskever, Vinyals, and Le 2014). There are two parts to this model: an encoder and a decoder, shown in Fig. 1. The encoder *E*, with encoding dimension *f*, takes as input a sequence and converts it into a vector of real numbers. This vector is then used as the initial state of the decoder, which constructs the goal sequence. seq2seq models have shown success in translation tasks (Bahdanau, Cho, and Bengio 2014; Luong et al. 2015) and text summarization tasks (Nallapati et al. 2016). For this reason, we viewed the problem of modeling protein evolution from parent to child as a translation problem. The seq2seq model in MutaGAN uses a bidirectional encoder (Schuster and Paliwal 1997), simultaneously evaluating the input sequence forward and backward to produce the optimally encoded vector.

### GANs

**Figure 2.**
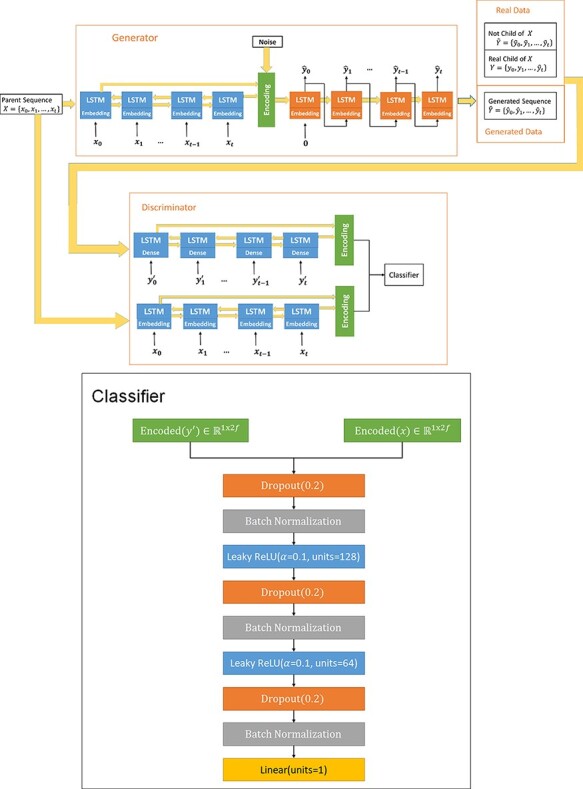
The MutaGAN framework’s architecture. The generator of the MutaGAN is a seq2seq translation deep neural network using LSTMs and embedding layers. The encoding layer uses a bidirectional LSTM. The output of the encoder is combined with a vector of random noise from a normal distribution *N*(0,1). The output of the decoder LSTM feeds into a softmax dense layer. An argmax function is then applied to select a single amino acid at each position, rather than a probability distribution. The discriminator uses an encoder with a slightly different structure from the encoder in the generator, but it uses the same weights. This is because an argmax function is not differentiable. Therefore, the first layer of the encoder in the discriminator is a linear dense layer with the same output size as the term embedding layer in the generator. This allows it to take as input, the output of the dense layer of the decoder in the generator. The weights of this dense layer are the same as those of the embedding layer, meaning that it produces a linear combination of the embeddings from the embedding layer of encoder. The discriminator takes in two sequences and determines whether the input sequences are a real parent–child pair or if they are not. The sequences that are not real parent–child pairs are a parent and generated sequences and two real sequences that are not parent–child pairs.

A GAN consists of two neural networks, a generator G and a discriminator D, that compete in a zero-sum game of the generator trying to fool the discriminator and the discriminator trying to distinguish real examples from generated examples. The traditional methodology for training a GAN alternates between training the discriminator and freezing the weights in the discriminator and training the GAN to generate sequences that the discriminator thinks are real. Typically, a GAN is trained to turn random noise into an output matching a known distribution. However, by conditioning the output of a GAN on a partially structured input, in addition to random noise, we can implement a conditional GAN (Mirza and Osindero 2014). The conditional GAN used in this paper is shown in Fig. 2. In the context of this work, our partially structured input was the parent protein sequence, which, once encoded, was combined with a random vector of noise. Because mutations are inherently stochastic, we identified a conditional GAN framework as the ideal model candidate for the use of a seq2seq model to generate numerous mutations given a single parent protein.

### Influenza

Influenza virus is an important human pathogen, causing significant annual morbidity and economic burden globally. In the USA, influenza contributes to over 30,000 deaths each year (Thompson et al. 2003, 2004; O’Brien et al. 2004). Human influenza A viruses are named based on the geographic location where the virus was isolated, the date of the isolate, and the identity of the two major surface proteins, hemagglutinin (HA) and neuraminidase (NA) (WHO 1980). There are eighteen distinct antigenic subtypes of HA (H1–18) and eleven distinct antigenic subtypes of NA (N1–11), with only H1N1 and H3N2 currently circulating in the human population (Kosik and Yewdell 2019; CDC 2022). While few influenza subtypes are circulating within the human population, a major concern has been the introduction of new, more infectious subtypes from animals, e.g. avian or porcine species. This so-called ‘species jumping’ has caused great concern due to the potential for a global spread, similar to the 1918 Influenza Pandemic (Webster 1999; Palese and Shaw 2007). A recent example highlighting this is the introduction of a new H1N1 strain in 2009 that was introduced from pigs in Mexico (World Health Organization (WHO) 2010; Hensley and Yewdell 2009; Michaelis, Doerr, and Cinatl 2009).

Current vaccine sequence selection is an inexact process based on recent field surveillance data to produce the seed stock for the next year. This approach has resulted in frequent mismatch between vaccine antigens and circulating virus. Another delay in vaccine production is caused by testing candidate vaccine sera against circulating strains. This antigenic characterization of influenza virus through serological interrogation with antibody-containing sera is crucial for virus titration, identifying new antigenic variants, vaccine strain selection, and epidemiologic studies. However, they are slow and costly and only address a small subset of potential variant viruses. Influenza virus presents significant challenges due to high virus polymerase error rate and reassortment of the segmented genome that result in a constantly changing protein landscape with antigenic drift and antigenic shift, respectively (Kawaoka, Krauss, and Webster 1989; Hensley et al. 2009; Medina and García-Sastre 2011; Imai et al. 2012; de Vries et al. 2013; Harding and Heaton 2018).

The small changes that occur from antigenic drift usually produce viruses that are closely related genotypically and antigenically, which can be illustrated on a phylogenetic tree. These close relations and similar antigenic properties often enable immune cross-protection (Tenforde et al. 2021). However, it has been shown that a single change in a particularly important antigenic location on the HA can also lead to the immune system unable to recognize the virus (Laver et al. 1979; Yewdell, Webster, and Gerhard 1979; Webster and Laver 1980; DeDiego et al. 2016; Li et al. 2016a; Lee et al. 2019). Antigenic drift results in influenza viruses existing as populations with a major genotype and multiple quasi-species (Kuroda et al. 2010; Lauring and Andino 2010). This mixed diversity can lead to vaccine mismatch and reduced protection (Tricco et al. 2013). Furthermore, antigenic drift over time leads to accumulation of changes and results in new antigenic characteristics that can evade the immune system or transmit between species. Current vaccine sequence selection is an inexact process based on recent field surveillance data to produce the seed stock for the next year (Perofsky and Nelson 2020). This approach has resulted in frequent mismatch between vaccine antigens and circulating virus. Further exacerbating vaccine failure is the process employed to evaluate immune response elicited by vaccination. Specifically, vaccinated animal sera are tested against the selected virus stock only, which limits the understanding of the breadth of immune response to other virus variants.

While deep learning has successfully been applied to genomics and biological sequence–related tasks, to date, there is no testable evolutionary forecasting model that predicts with high confidence which virus genotypes will emerge and circulate annually. There has been work studying the mutation of viruses and viral escape using deep learning (Neher, Russell, and Shraiman 2014; Hie et al. 2021) or using statistical methods to model fitness (Bush et al. 1999; Luksza and Lässig 2014, Morris et al. 2018). However, these do not identify direct parent to child relationships of all sequences nor predict future progeny sequences of all parent sequences. Furthermore, other models developed to predict virus evolution from training data focused on identifying a single, most likely clade emerging (Neher, Russell, and Shraiman 2014; Neher et al. 2016). This method is not capable of projecting mutations at multiple locations. While understanding clade emergence is important, this approach does not provide insight into the likelihoods of individual site mutations and, importantly, cannot be applied to intrahost evolution. To provide a clearer picture of the different possible evolutionary trajectories, we developed a novel machine learning framework that uses broad historical training data to predict all likely mutations in an influenza virus genome segment sequence.

### Contributions

In this paper, we present MutaGAN, a novel deep learning framework that utilizes GANs and seq2seq models to learn a generalized time-reversible evolutionary model. We do this by building a model capable of generating mutations for a given input parent sequence, replicating key aspects of the phylogenetic tree. We then demonstrate its capability of accurately modeling the mutations observed in phylogenetic data of the H3N2 influenza A virus HA protein. This process is the first deep learning model that attempts to model and predict the evolution of a protein with minimal human input and no human supervision.

## Material and methods

### MutaGAN

The core of our model was a seq2seq translation deep neural network, which formed the generator in the GAN. The seq2seq encoder *E* takes as input a sequence of length *N*, with a dictionary size *d*, and converts it into a vector of length *m*, $E:{\rm{ }}{\mathbb{R}^{N{\rm{ x }}d}} \to {\rm{ }}{\mathbb{R}^{1{\rm{ x }}m}}$, analogous to the embedding layer. The embedding layer of this network was created using a biological language of 3-mers of amino acids with a sliding window with step size of 1. For example, the sequence of amino acids *MKTIIALSY* is transformed into *MKT KTI TIL ILA…*. The output of the decoder LSTM is fed into a softmax dense layer (Fig. 2). To achieve our goal of a model that can generate different sequences for a given parent, the element-wise random noise vector sampled from a standard normal distribution was combined with the output of the encoder.

The structure of the encoder in the discriminator is slightly different from that of the encoder in the generator, but it uses the same weights. An embedding layer requires the input to be in the form of a single integer, representing a discrete input. However, the output of the generator at each time step is a probability vector with dimension ${\mathbb{R}^{d{\rm{ x }}1{\rm{ }}}}.$ This cannot be transformed into an integer with the argmax function because the argmax function is not differentiable, meaning that it does not allow for backpropagation to train the generator. Therefore, a modified encoder takes as input a vector with dimensions ${\mathbb{R}^{d{\rm{ x }}1{\rm{ }}}}$, with the first layer of the modified encoder being a linear dense layer with an output of $m\,$and no bias term. The weights of this dense layer are the same as those of the embedding layer, meaning that it produces a linear combination of the embeddings from the embedding layer of the encoder for parent sequences in the discriminator and the encoder in the generator. The generated sequences were fed into this encoder as softmax outputs of the generator, and the real sequences were fed in as one-hot encoded sequences.

The architecture of the discriminator was built using code available on https://github.com/DanBAPL/MutaGAN, with the final layer of the discriminator being a sigmoid function. This includes the loading of the pretrained autoencoder weights. Because the two encoders used the same bidirectional LSTM, the weights for that in the two encoders were automatically shared once they were loaded into the parent encoder.

The embedding layer in the model had a dimension of 250 and allowed for 4,500 tokens. Unknown tokens are given the value ‘[UNK]’. We did not have a problem in generating sequences that had unknown tokens on input, as the sliding window with an overlap provided cover. The bidirectional LSTM encoder had 128 units, making the encoding dimension $f$ = 512 (both cell and hidden states for forward and backward LSTMs), and the LSTM decoder had 256 units. The discriminator consisted of concatenated hidden and cell states of the LSTM encoder and modified LSTM encoder, as described earlier. This was fed into fully connected layers of three sets of dropout (20 per cent), batch normalization, and a dense layer. The first two dense layers had 128 and 64 dimensions and used a leaky ReLU activation function with $\alpha = 0.1$. The final dense layer was a linear activation function with Dimension 1.

### Dataset

For this work, the influenza virus was chosen as an ideal test case for this deep learning framework because it is a significant human pathogen that changes rapidly, with new strains emerging annually, and global surveillance efforts have generated large amounts of publicly available genomic data (Wohlbold and Krammer 2014). The surface proteins HA and NA of influenza virus enable virus entry into cells and are the primary immune epitopes that elicit antibodies, making them of particular interest for vaccine development (Wohlbold and Krammer 2014).

### Database curation

Influenza virus HA sequences were downloaded from the National Center for Biotechnology Information’s (NCBI) Influenza Virus Resource (IVR) (Bao et al. 2008). Utilizing Bash text parsing methods including *awk* and *sed*, the dataset was curated to gene sequences from the influenza A type and H3N2 subtype that were obtained from human hosts between 1968 and 2017 and validation data included strains from 2018–19. Only sequences dated between 1 January 2018 and 31 December 2019 were used for the validation dataset. Duplicate records were removed using the ‘isolate_name’ and ‘isolation_date’ metadata attributes as a unique identifier. When a duplicate identifier was encountered, the first record within the IVR database was kept and the remaining records were discarded. Additionally, only isolates that had full-length HA segments present in the dataset were kept. Of note, during curation, twenty-two isolates from swine or avian hosts remained in this dataset (Supplementary Table S1). When completed, the curated sequence dataset contained 6,840 unique records of H3N2 influenza virus unique sequences.

### Phylogenetic tree generation

**Figure 3.**
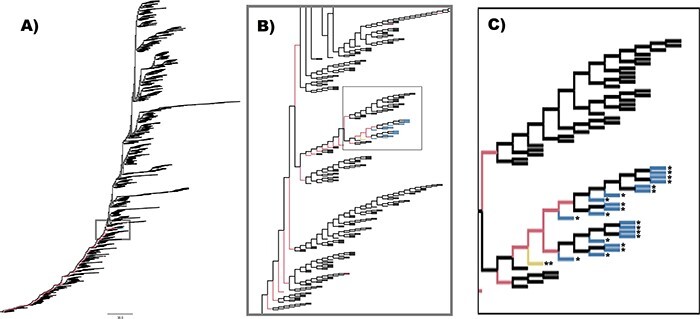
Topology of RAxML tree used to build parent–child pairs. The topology of the maximum likelihood tree created from 6,840 H3N2 sequences is shown in (A). The ancestral sequences of each internal node in this tree were used to form the 13,768 parent–child pairs used to train the seq2seq generator of the GAN framework. An outlying group, containing twenty-two sequences, was identified as coming from swine or avian hosts, and those sequences are indicated in blue and with *. One of these twenty-two sequences was from the group of 155 parent-child pairs with a Levenshtein distance >10 and is indicated in yellow and with **. The region surrounding that outlying group (gray box) is expanded in the inset (B) and further expanded in the insert (C), where it can be seen that the majority of the parent–child pairs removed for high Levenshtein distance in non-human hosts come directly off the backbone of the phylogenetic tree leading to the outlying group in blue. This trend continues back to the root of the tree.

For input into the seq2seq GAN framework, phylogenetic reconstruction was performed using the nucleic acid sequences of the 6,840 HA sequences. All DNA sequences were aligned using Multiple Alignment using Fast Fourier Transform (v.7.471) (Katoh and Standley 2013) and trimmed to the coding region. The final maximum likelihood (ML) tree was made using RAxML (v.8.1.1) (Stamatakis 2014) with a generalized time reversible model, gamma model of rate heterogeneity, and ML estimate of the alpha parameter. The final tree, as shown in Fig. 3, was visualized using FigTree (v.1.4.4) (Rambaut 2017) with some custom post-processing.

### Dataset creation

After rooting the final ML tree to isolate ‘influenza A virus A/Hong Kong/1/68(H3N2)’, Marginal ancestral sequence reconstruction was performed with RAxML using the General Time Reversible model of nucleotide substitution with the gamma model of rate heterogeneity. Parent–child relationships were generated using the Bio.Phylo package in BioPython (Cock et al. 2009) and were limited to single steps between phylogenetic tree levels such that each parent had exactly two children. One parent–child pair was generated for each of the 13,678 edges within the final binary tree. Because phylogenetic tree generation requires removal of duplicate nucleotide sequences prior to evolutionary modeling, there was a concern of providing an information bias of evolution towards the ancestral sequences (i.e. internal nodes) and away from sequences acquired through genomic surveillance (i.e. leaf nodes). To mitigate this bias, leaf nodes that had a nucleotide sequence matching to multiple records within the IVR database were inserted back into the dataset as duplicate parent–child pairs. As an example, if a leaf node’s sequence was observed four times in IVR, there would be four identical parent–child pairs inserted into the dataset. Upon completion, the number of parent–child pairs was increased to 17,218 within the formatted dataset. Each nucleotide sequence was translated to amino acids for representation learning of the HA protein by the MutaGAN framework.

The training and test datasets were formed by splitting a list of the compiled unique parent sequences in a random 90/10 split. The result was 1,451 unique parent sequences in the training dataset and 156 unique parents in the test dataset. There were a total of 15,699 parent–child pairs in the training dataset and 1,519 parent–child pairs in the test dataset. A total of 150 sequence pairs (0.96 per cent) were removed from the training dataset, and 11 sequence pairs (0.72 per cent) were removed from the test dataset in which the amino acid Levenshtein distance (see the Generator evaluation section for description) was ten or greater to prevent parent–child pairs that were excessively unrelated, either from sampling bias or mistaken sequence inclusion. This removal had the effect of isolating an outlier group identified within our phylogenetic tree that appeared as a result of a small number of sequences not being removed during the pre-phylogenetic filtering process. Because the phylogenetic tree was created using the virus gene sequences and synonymous mutations do not lead to amino acid mutations in proteins, the corresponding parent–child protein pairs could be identical. Of the 1,451 unique parents in the training dataset, 103 parents (7.10 per cent) only had child sequences that were identical to the parents, while 567 (39.08 per cent) had only one unique child. For a measure of parent–child diversity, the training set contained 5,048 parent–child pairs where the child’s sequence differed from its parent. Matching parent–child pairs were removed from the training dataset. In the test set, all instances of matching parents and children were removed, leaving 433 parent–child pairs with 141 unique parent sequences. The test dataset only contained pairs in which the parent and child sequences were different.

The validation dataset was built using the same phylogenetic tree construction process as the training and test datasets, but using only data from 2018 and 2019. As a result, it is a temporally distinct dataset from the training and test dataset, separated by a full year. This dataset had 3,260 parent–child pairs. Of these 3,260 pairs, ten (0.3 per cent) were removed for having a Levenshtein distance greater than 10. The remaining 3,250 pairs contained 1,807 (55.6 per cent) of sequences where the parent and child were not the same. There were 561 unique parent sequences, and 287 (51.2 per cent) had only one child sequence.

### Generator evaluation

The most important metric for assessing quality of the generated sequences is whether they were able to produce the mutations observed in the data. However, missing an observed mutation does not necessarily mean that the generator did not correctly predict possible mutations and only that it did not correctly predict all observed mutations. It is possible that predicted mutations would have likely occurred but just were not included in our subset.

We identified mutations in our sequences by measuring the Levenshtein distance between parent and child sequences. By using the Levenshtein distance, we were able to account for insertions and deletions as well as mutations (Levenshtein 1966), and by using the diff-match package from Google, we were able to identify where changes were made between two sequences (Fraser et al. 2018). The diff_cleanupSemantic function in the diff-match-patch package was used to identify where changes were made between parents, real children, and generated children. A list of all child mutations was created for each parent and compared to each parent’s generated children. A mutation was counted as correct if change occurred in the generated child that was identical in amino acid and location as observed in the parent’s real children. A partially correct mutation was defined as an amino acid change in the generated child that was identical in location to any mutation in a real child of that parent but differed in amino acid type. False mutations were defined as mutations that were predicted but non-existent in the real children, and missed mutations were defined as mutations observed in at least one of the real children of the parent but not in the generated child.

In addition to the Levenshtein distance, we use four metrics for evaluating the performance of the generator: known mutation location rate, amino acid mutation frequency, true-positive rate, and weighted true-positive rate.

#### Known mutation location rate

The first and our primary evaluation metric is the percent of parent sequences for which MutaGAN was able to generate at least one known mutation. This mutation must be a known mutation for that parent sequence, not any random parent. We refer to this as the known mutation generation rate. This can be extended to mutations that are in the correct location but incorrect change. This is the known mutation location rate. This is calculated as follows: for the ${i^{{\rm{th}}}}$ parent sequence with known child sequences, ${C_i} = \left\{ {{c_0},\,{c_1}, \ldots } \right\}$ and set of known mutations ${{\mathcal M}_i} = \left\{ {{m_0},{m_1}, \ldots } \right\}$. For a given input parent sequence used to generate $k$ potential sequences, we can create a set of potential mutations ${\mathcal M}_i^{^{\prime}} = \left\{ {m_0^{^{\prime}},\,m_1^{^{\prime}}, \ldots } \right\}$


(1) $$p = \frac{1}{N}\mathop \sum \limits_i^N \begin{cases}1 & {\rm if}\ \left|{M_{i}^{^{\prime}}} \cap {M_{i}} \right| \gt 0 \\& {0\,{\rm{\;else\;}}}\end{cases},$$

where $N$ is the total number of parent sequences.

#### Amino acid mutation frequency

Frequency of amino acid mutations was calculated to evaluate the similarity of the mutation profiles between MutaGAN and the ground truth data (Equation 2). For each mutation within a given set of mutations, ${a^{\left( p \right)}}$ is the amino acid of the parent, ${a^{\left( c \right)}}$ is the amino acid of the child, and ${x_{{a^{\left( p \right)}}{a^{\left( c \right)}}}}$ is the count of all mutations observed from one amino acid to another. The amino acid mutation frequency is the value ${x_{{a^{\left( p \right)}}{a^{\left( c \right)}}}}$ divided by the total number of recorded mutations. This provides information on the frequency of mutating one amino acid to another:


(2) $${f_{{a^{\left( p \right)}}{a^{\left( c \right)}}}}\, = \,\frac{{{x_{{a^{\left( p \right)}}{a^{\left( c \right)}}}}\,}}{{\mathop \sum \nolimits_{a \in A} \mathop \sum \nolimits_{a{^{^{\prime}}} \in A} {x_{{a^{\left( p \right)}}{a^{^{\prime}\left( c \right)}}}}\,\,}}.$$

#### *True*-*positive rate*

There are two ways of evaluating whether the generated sequences contain the known mutations. The first is to consider all the mutations for a known parent and determine whether the generated sequences contain those mutations. This can be measured similar to the standard true-positive rate formula, and thus, we will refer to it as the standard true-positive rate,


$$\begin{aligned}& {\text{TPR}} \\ & = {\text{\;}}\frac{{{\text{\;number of correct\;mutation}}}}{\begin{aligned}{\text{number of incorrect mutation}} & + {\text{number of no mutation}}\\ \phantom{\text{\;number of incorrect\;mutation}} & + {\text{number of correct\;mutation\;}}\end{aligned}}.\end{aligned}$$

This is useful in determining how well the variety of mutations are reflected in the generated sequences. However, it does penalize sequences with multiple known mutations unless those generated sequences contain all known mutations, which is not something we want, as we want these mutations distributed across sequences to be proportional to how they would appear in the sequencing data. Take, for example, a parent sequence ACFKLM has two children, ACFHLM and ACFHIM. If MutaGAN generates 100 sequences, fifty of them are exact matches to Child 1 and fifty of them are exact matches to Child 2, the true-positive rate would be 50.0 per cent because only half of known mutations appear across all generated sequences. If all generated sequences were ACFHIM, the true-positive rate would be 100.0 per cent because all known mutations appear in all children. However, we want our generated sequences to be more similar to the former scenario than the latter.

Therefore, we will also use a variation on the standard true-positive rate in which the number of times a mutation was made or missed is ignored, paying attention only to whether it happened across a given parent. We refer to this as the sequence true-positive rate because it focuses on the presence of mutations across a given parent sequence. The sequence true-positive rate is calculated the same way as the standard true-positive rate is calculated, with one additional step: a unique list of all the mutations is made for sequences generated for each parent, rather than using the mutations made for each generated sequence.

#### Weighted true-positive rate

To account for different levels of similarity between any two amino acids when evaluating mutational errors, Sneath’s index (Sneath 1966), a percentage representation of the number of dissimilar comparisons of amino acids along 134 categories of activity and structure, was incorporated into a calculation for weighted accuracy. For this analysis, we removed the prediction of the ambiguous amino acid designation (X). Additionally, we set the lower limit on the allowable similarity to 0.85 to prevent over rewarding when calculating weighted averages. Only eighteen different amino acid pairings with Sneath’s Index have similarities ≥0.85 and were included, while all other comparisons were set to 0. This means there are only eighteen different types of mistakes for which partial credit can be awarded. To calculate the weighted accuracy, each mutation found in the set of generated children was weighted using thresholded Sneath’s index, $S$, and averaged across the entire table of predicted mutations, $A$, where the columns are the predicted amino acid and the rows are the expected amino acid, as calculated in Equation 3,


(3) $${avg}_{w} = \frac{{sum}\left( {\left[ {S \otimes \left( {S \ge 0.85} \right)} \right] \otimes A} \right)}{{sum}\left( A \right)},$$

where $ \otimes $ is an element-wise multiplication of two matrices with the same dimensions.

### Experiment

In this section, we present the experiment we designed to train and test MutaGAN.

### Setup

The phylogenetic tree reconstruction took place on a 16 processor 64 GB RAM compute node running Ubuntu. RAxML tree optimization and ancestral reconstruction took roughly 14 days to complete. The models were built, and training and testing was implemented in Python version 3.8.8 using the libraries Tensorflow version 2.6.0 (Abadi et al. 2016) and Keras version 2.6.0 (Chollet 2015) on four GeForce GTX 1080 Ti graphical processing units. Additionally, metrics were calculated using the functions in the package scikit-learn version 0.24.1(Pedregosa et al. 2011), the diff-match-patch package (Fraser et al. 2018), and the natural language toolkit package (Bird, Klein, and Loper 2009).

#### Model training

The maximum number of words included in the embedding layer was 4,500, which was selected by rounding up the number of unique 3-mers found in our dataset. Additionally, we selected an embedding size of 250. The encoder portion was a bidirectional LSTM with 128 nodes, resulting in a state vector of 512 being passed to the decoder, which was a unidirectional LSTM. Generator pretraining was performed on the training dataset and tested on the test dataset. This was performed using the Adam optimizer (Kingma and Ba 2014) with a learning rate of 0.01 until the model reached a stable state. It was set to train for 72 epochs, but converged far before that.

GAN training occurred in two stages based on batch size, for a total of 350 epochs. In both stages, we selected the Adam optimization algorithm and the learning rate for the generator was 1 × 10^− 3^ and the discriminator was 3 × 10^−5^. The learning rate for the discriminator was chosen to avoid mode collapse. The first 200 epochs of the model were trained on a batch size of 32 with the discriminator for five epochs and the generator training for five epochs. The last 150 epochs of the model were trained on a batch size of 45 with the discriminator and the generator training for five epochs each.

Typically, the discriminator is only meant to help the generator create realistic data, but the MutaGAN discriminator has the added goal of making sure that the generated sequence is a possible child of the parent. Therefore, we modeled our approach after Reed et al. (Reed et al. 2016) and created three types of sequence pairs to train the discriminator. The first pair type is real parents and real children, as determined from the phylogenetic model. The second is real parents and generated children. The third is real parents and real non-children. The purpose of the third pair is to ensure that the model learns to differentiate between related and unrelated sequences in the context of evolution. Ten thousand training records of the third type were generated by randomly pairing unrelated parent and child sequences with a Levenshtein distance >15. A lower bound of fifteen was selected because we wanted to avoid unrelated parent child pairs that could be too close, as those could be parent–child pairs that were not sequenced and therefore not observed. As a result, we could model sequences that were similar and real, but not directly related.

To optimize performance of the model, our framework deviated from previously published methods in a number of ways. The MutaGAN seq2seq model was pretrained prior to input into the GAN using teacher forcing (Williams and Zipser 1989), so the generator’s decoder also contained a similar embedding layer with 4,500 words and an embedding size of 250. The loss function was the standard sparse categorical cross entropy loss function.

The initial version of the GAN used a binary cross-entropy loss function (Equation 4),


(4) $$L = \frac{1}{N}\mathop \sum \limits_{n = 1}^N - \left( {{y_n}\log \left( {{p_n}} \right) + \left( {1 - {y_n}} \right)\log \left( {1 - {p_n}} \right)} \right).$$

However, early iterations of our model using this loss function were characterized by mode collapse, where the generator produces an unvarying child sequence given a single parent sequence. To resolve this problem, the loss function was switched from binary cross-entropy to Wasserstein loss


(5) $$L = \frac{1}{N}\mathop \sum \limits_{n = 1}^N {y_n}*{\hat y_n}.$$

where ${y_n}$ is the ground truth value, either 1 or −1, and ${\hat y_n}$is the predicted value, and the final layer of the discriminator into a linear activation function (Arjovsky, Chintala, and Bottou 2017). The loss of the generator is the sum of the Wasserstein loss and the sparse categorical cross-entropy of generating the child sequence.

In a variation from Reed et al. (2016), we used sequences generated by the initial GAN as additional negative examples in training the discriminator of the final model to prevent the model from drifting too far off course as a form of experience replay, similar to an approach used in deep reinforcement learning (Lin 1992). The initial GAN created a high proportion of generated children with a Levenshtein distance >300 (Supplementary Fig. S1). Using this model to generate 10,000 children from randomly selected parents, with replacement, and removing pairs where the Levenshtein distance was <15, we were left with 8,550 parent–child pairs. These sequences were used for experience replay. The distribution of the Levenshtein distances of the sequences for both the fake parent–child pairs and the experience–replay pairs is shown in Supplementary Fig. S1 in a stacked histogram, with the real parent and real non-child sequences in blue and the real parent and generated sequences from the failed model in orange.

As a baseline comparison model, we used a Monte Carlo simulation with the training data acting as our source of the historical statistics to create a fixed probability model. For this Monte Carlo simulation, we create three distributions from the training data: number of mutations, location of mutation, and amino acid change. For the distribution of the number of mutations, ${p_{{\rm{num}}}}$, we used the number of mutations in the training data and sampled from that. For the distribution of the location of the mutation, ${p_{{\rm{loc}}}}$, we sampled from the distribution of the locations of known mutations in the training data and then randomly sampled from a set of $\left\{ { - 2,\, - 1,\,0,\,1,\,2} \right\}$ to perturb that location to provide some additional variability. For the distribution of the amino acid change, ${p_{{\rm{aa}}}}$, we used the distribution of amino acid changes in the set of known mutations in the training data. Therefore, the process of generating a new sequence using the baseline method is as follows:

1.
$n \sim {p_{{\rm{num}}}}$

2.
$l \sim {p_{{\rm{loc}}}}$ until there are $n$ unique $ls$
3.
${\hat y_i}\, \sim \,{p_{aa}}\left( {{x_i}} \right)$ for the amino acid ${x_i}\,$at location ${l_i}$ for $i = 1, \ldots ,n$.

As with MutaGAN, 100 sequences were generated for each parent in the validation dataset. The results for the baseline model are shown in Table 1.

**Table 1.** The results and metrics of the MutaGAN model and the baseline model on the validation data.

Metric	MutGAN performance on the validation dataset	Baseline model on the validation dataset
Known mutation generation rate	72.8%	44.1%
Known mutation location rate	77.8%	77.1%
Median Levenshtein distance		
Generated vs child	4.00 ($\mu = 4.83,$$\sigma = 4.12$)	1.00 ($\mu = 1.62,$$\sigma = 1.10$)1.00
Generated vs parent	4.00 ($\mu = 5.10,$$\sigma = 4.14$)	($\mu = 1.69,$$\sigma = 1.14$)
Average difference in amino acid mutation frequency		
Generated vs training	3.2 × 10^−3^	9.1 × 10^−4^
Generated vs validation	2.0 × 10^−3^	1.1 × 10^−3^
True-positive rate		
Standard (weighted)	23.6% (60.5%)	0.1% (32.3%)
Sequence level (weighted)	25.3% (67.0%)	3.1% (79.2%)

## Results

After the model was trained, we generated 100 child sequences for each of the 562 unique parent sequences in the validation set. After removing overlap between parents from the training and validation set, there were 536 unique parents. The process of generating the 53,600 sequences took approximately 30 min. We then discarded 1,032 sequences with >sixty amino acid differences from their parent sequences, a non-biological artifact that we are treating as noise. A change of this magnitude corresponds to 10 per cent of the overall protein structure and is highly improbable to have occurred by chance within a single evolutionary step on the timescale with which the phylogenetic model was created. There were two parent sequences we were unable to generate viable children for, corresponding to a 0.36 per cent failure rate. MutaGAN’s performance is summarized in Table 1. The median Levenshtein distance between parent and observed child amino acid sequences within the validation dataset was 1.00 ($\sigma = 1.06)$. The median Levenshtein distance between the generated sequence and the closest child sequence of the input parent in the validation dataset is 4.00 ($\mu = 4.83,\,\sigma = 4.12)$, compared to 4.00 ($\mu = 5.10,\,\sigma = 4.14)$ between the generated and parent sequence (Supplementary Fig. S2). The generated sequences were marginally closer to the child sequences, but still very close, to the parent sequences. This indicates that the model is augmenting its input to account for the learned model of protein evolution.

The known mutation generation rate was 72.8 per cent, as 390 of the 536 parent sequences had at least one observed mutation augmented onto it within its generated child sequences. Of the parent sequences that MutaGAN did not correctly identify a mutation as observed in the ground truth, there were twenty-seven(5 per cent) sequences for which MutaGAN produced a mutation in the correct location but with the incorrect amino acid. Therefore, the known mutation location rate is 77.8 per cent.

**Figure 4.**
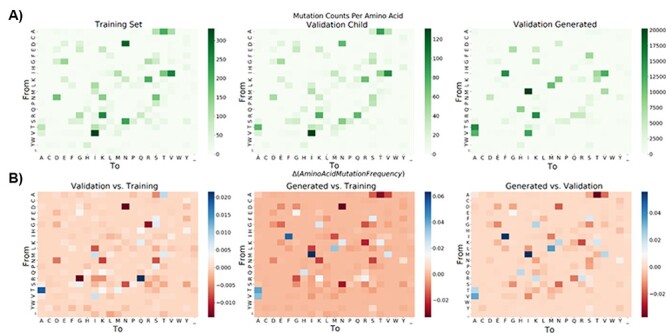
Amino acid mutation profiles with respect to amino acid types. For the training, validation, and generated child sequences, total counts for each amino acid mutation from parent to child are displayed in (A). Amino acid ordering was determined using R’s hclust function on the training data and kept consistent throughout both (A) and (B). Differences in amino acid mutation frequency between the training, validation, and generated datasets were calculated and are visualized in (B) using Equation 2

Because amino acids range in biochemical and physical similarities, it is important to look closer at the actual mutations that are made or missed, especially because many of the extra mutations correspond to common biological mutations between functionally similar amino acids. Mutation profiles by amino acid are provided in Fig. 4. A side-by-side comparison of the mutational profiles is made across the training, validation, and MutaGAN-generated amino acid sequences with respect to the parent input sequences. MutaGAN’s amino acid mutational profile is strikingly similar to that of both the training and validation datasets, indicating that the model has learned a measure of biological significance in the biophysical and chemical properties of amino acids. To assess if MutaGAN’s generated amino acid mutation profile more closely resembles the training set over the validation set or vice versa, the average difference in amino acid mutation frequency was calculated for the ‘Generated vs Training’ and ‘Generated vs Validation’ delta mutation profiles (Fig. 4B). This measure of distance was calculated to be 3.2 × 10^−3^ and 2.0 × 10^−3^, respectively. Importantly, the MutaGAN generated mutation profile shows changes in mutation frequency for specific amino acids that more closely resembles the validation set when compared to the training set (Fig. 4A). In particular, it is observed that there are higher proportions of threonine (T)→lysine (K), threonine (T)→isoleucine (I), arginine (R)→lysine (K), and glycine (G)→aspartic acid (D) mutations and lower proportions of alanine (A)→valine (V), glycine (G)→arginine (R), and alanine (A)→threonine (T) within the ground truth validation data as compared to the ground truth training data. For these same amino acids, MutaGAN’s mutational profile shows the same trends.

The results in Table 1 indicate that the MutaGAN model and the baseline model were able to comparably generate mutations at sites known for mutations in the validation data, with the known mutation generation rate of models being 77.8 per cent and 77.1 per cent, respectively. However, the known mutation generation rate for MutaGAN was 72.8 per cent vs the baseline rate of 44.1 per cent. Additionally, the true positive rate (TPR) scores are significantly higher for MutaGAN. While the median Levenshtein distance and the average difference in amino acid mutation frequency were lower for the baseline model, this is expected. Additionally, this indicates that MutaGAN, while capable of capturing a similar profile of mutation locations, is capable of making mutations outside the historical distributions that match the validation data.

The most prominent amino acid mutations that were made by MutaGAN that were not seen frequently in either the training or validation data are glutamine (Q)→arginine (R), asparagine (N)→aspartate (D), asparagine (N)→serine (S), and histidine (H)→asparagine (N) (Fig. 4B). Interestingly, Arginine is the second most favorable amino acid mutation from glutamine behind glutamate (Barnes and Gray 2003). Aspartate and serine are the most favorable amino acid mutations from asparagine alongside histidine, and asparagine is the second most favorable mutation from histidine behind tyrosine. Another frequently incorrect MutaGAN mutation of note is serine (S)→proline (P). Serine, when present on a protein’s surface, often forms hydrogen bonds with the protein’s backbone and effectively mimics proline (Barnes and Gray 2003). In accordance, the four locations that MutaGAN incorrectly mutated the HA protein from a serine to a proline were at amino acid positions 143, 198, 199, and 227 within the HA1 chain, all of which are located on protein’s surface.

As an artifact of the phylogenetic analysis, a small but noticeable portion of child sequences within the training, test, and validation datasets contained the ambiguous amino acid symbol ‘X’ at some location within its sequence. The appearance of ‘X’ in a child sequence created the appearance that a parent amino acid could mutate to ambiguity. However, MutaGAN never mutated a parent amino acid to ambiguity (Fig. 4A). This is likely due to the fact that of all the amino acids in the training dataset, only 3.35 × 10^−3^ per cent were ‘X’, meaning that there were too few examples of ‘X’ for it to learn it.

**Figure 5.**
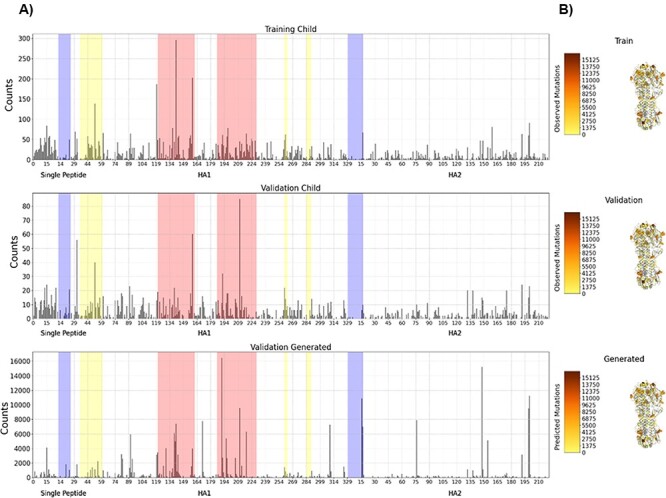
Amino acid mutation profiles with respect to HA protein locations. For the training, validation, and generated child sequences, total counts of mutations observed across the entire length of the HA protein segment are displayed in (A), indicating the signal peptide, HA1 (head), and HA2 (stalk) regions of the full HA protein. The most highly variable regions are highlighted in salmon, the third and fourth highlighted regions. Regions of lesser, but still significant variability, are highlighted in yellow, the second, fifth and sixth highlighted regions. Particularly conserved regions are highlighted in blue, the first and last highlighted regions. In (B), a diagram of the H3 HA structure (PDB: 4GMS) is colored by this mutation frequency, with the positions with the fewest mutations in yellow to the positions with the most mutations in brown. Positions with zero observed mutations across each dataset are colored gray. Residues are displayed as spheres for positions with mutation frequencies above 30 per cent of the maximum position for each of the three datasets. These 30 per cent threshold lines are also plotted in (A).

The overall mutation location profile of historical H3N2 influenza virus HA proteins was well reproduced by MutaGAN (Fig. 5). Supplementary Figure S3 shows the same plot on the test data for comparison purposes. The most highly variable regions identified in the training and validation datasets (HA1 amino acid indices 120–160 and 185–228) were also the most mutated regions by MutaGAN. Regions of lesser, but still significant, variability were also identified by MutaGAN in accordance with the historical H3N2 data observed in the training and validation sets of Fig. 5 such as HA1 residue regions (i.e. amino acid indices) of 45–59, 259–262, and 273–278. Regions of historical conservation were accurately preserved by MutaGAN, most notably the HA1 residue region of 11–24 and the HA2 residue region 1–16. Of the top ten most frequently mutating positions in the training dataset, MutaGAN’s only had one within its own top ten (Position 121). Between the validation set and MutaGAN-generated set, there were no overlaps in the top ten most frequently mutated positions. However, for many of the most frequently mutating amino acid locations within the training and validation sets, the location was one or two positions away from a commonly mutated position in the MutaGAN-generated sequences. For instance, HA1 residues 142, 160, and 193 were in the top ten most frequently mutated positions in the training and validation sets. HA1 residues 145, 159, and 192 were in the top ten most frequently mutated positions by MutaGAN. This phenomenon is worth noting because of the closeness, but the biological significance is not readily apparent without a deeper analysis of the HA protein structure. In looking at the structure of the HA protein more closely (Fig. 5B), it is clear that the concentration of the most frequently mutated position for both the training and validation data sets occurs outside of the protein structure, principally on the outer surface of the HA1 domain toward the host-recognition regions. It is well understood that the frequent mutation of amino acids at these locations increases the influenza virus’s likelihood of evading host antibodies during infection. The majority of the MutaGAN-generated mutations also occur in these same regions but across a notably larger number of residues on the protein surface. This finding alludes to the ability of this framework to illuminate localized function across varying regions of the overall protein structure, but further simulations must be performed to investigate the functional effects of the MutaGAN-generated mutations.

We can also examine whether the model is simply repeating the same mutations or whether it is selecting mutations based on the input sequence. We can determine this by comparing the counts of mutations for generated sequences, shown in the bottom graph of Fig. 5, which uses sequences from 2018 to 2019 and Supplementary Fig. S3, which uses data similar to the training dataset from 1965 to 2017. These figures appear substantially different, especially in all seven areas of interest. This indicates that the model is not simply generating the same mutations regardless of the training data, but generating new mutations based on the input.

**Figure 6.**
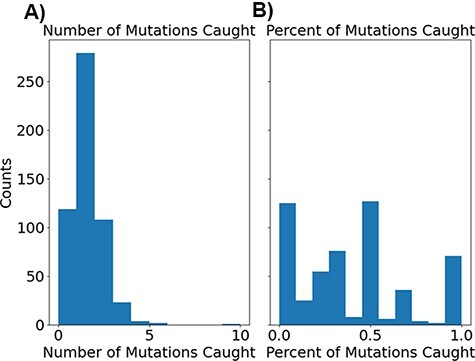
Histograms showing the distribution of correct mutations as both total counts (A) and percentage of total recorded mutations (B).

While recognizing that the phylogenetic tree does not capture the entire breadth of mutations that occurred during the entire evolution of the influenza virus, MutaGAN’s performance was evaluated with respect to this tree as our closest proxy of its ability to mimic the virus’s evolutionary landscape. The number of observed mutations reproduced for each parent is visualized in Fig. 6 and shows that most generated children contained at least one observed mutation, while a smaller number contained more.

As a measure of the standard true-positive rate, each generated child’s sequence contained 23.6 per cent of observed mutations between its parent and real children (the standard true-positive rate comparing the generated and real child sequences). Using Sneath’s index (Sneath 1966) to get a deeper assessment of the closeness of MutaGAN’s predictions, we find that it has a weighted true-positive rate of 60.5 per cent, calculated using Equation 3, to compare the generated and real child sequences. This large increase from an unweighted standard true-positive rate of 23.6 per cent to 60.5 per cent indicates that a majority of the mutations found within the real child sequences are similar in biochemical and physical properties to the amino acids MutaGAN used in those locations. When we use the sequence true-positive rate, we find that the unweighted sequence true-positive rate is 25.3 per cent and the weighted sequence true-positive rate is 67.0 per cent.

For 300 parent sequences (53.6 per cent), our model generated the same child sequence in each of the 100 iterations, regardless of the noise, using the distribution *N*(0,1) (Supplementary Fig. S4). While this behavior might appear to be mode collapse, in the ground truth validation data, the phylogenetic tree had only one child for 397 (71 per cent) of the parent sequences as a direct result of using the amino acid rather than nucleotide sequences and masking synonymous mutations. Of these 560 parent sequences whose generated children exhibited mode collapse–like behavior, 287 (51.25 per cent) also had only one child in the ground truth phylogenetic tree data. Since these proportions of parents with only one child are similar in both the generated results and the validation dataset, and notably larger than the 39.1 per cent in the training dataset, and there is a large overlap in sequences with only one child in the generated results and validation dataset, it appears that the mode collapse is only partially responsible. This is further supported by the standard and sequence level true-positive rates, which indicate a defaulting to one type of known mutation, rather than generating a variety of them. Future work, such as operating directly on nucleotide sequences, could help reduce the impact of mode collapse on the sequence generation.

## Discussion

### Accurate mutation forecasting from protein sequences

The MutaGAN framework presented here is the first method to utilize a GAN to accurately reproduce and optimize full-length proteins above 300 amino acids in length with no structural information provided to the model beyond amino acid sequence. The accurate reproduction of the mutation profiles of the HA protein with specificity of both the types of amino acids changed (Fig. 4) and locations most likely for persistence (Fig. 5) demonstrates the potential of this method to be used as a tool for forecasting the genetic drift or shift that occurs during the outbreak of the influenza virus. Our findings indicate that the sequence augmentation strategies deployed by MutaGAN optimize its input toward the most successful patterns observed during the evolutionary history of a protein. Because this framework is agnostic to the type of phylogenetic tree and protein type used to generate parent–child pairs, the extension of these methods to new proteins and organisms (e.g. the NA protein for influenza and the dengue virus) is ripe for exploration.

The ability of MutaGAN to learn and optimize mutations for persistence within a population lends itself well to protein engineering applications. As demonstrated in Figs 4 and 5, the unique nuances of change within a protein population are capable of being captured without any additional expert knowledge being provided to the model beyond a list of parent–child pairs for training tailored to a specific protein. The observation that MutaGAN inserts mutations that are biologically relevant, even when not observed in the ground truth data, poses the question of whether these mutations produce energetically favorable protein conformations with increased fitness within the evolutionary landscape. Future work could pair computational protein modeling with this framework for a deeper analysis of the MutaGAN-generated sequences for improved forecasting of population-level mutation propagation. With direct ties to the public health domain, by measuring the conformational protein favorability of MutaGAN-generated sequences and analyzing their similarity to currently circulating pathogenic sequences, public health officials could assess the threat of potential mutations against vaccine evasion and improve the design of future treatments or vaccines.

In addition to identification of persistent mutations in a population, MutaGAN has potential to predict novel mutations. Previous models utilizing evolutionary information are able to identify historical sequences that may give rise to future clades (Neher, Russell, and Shraiman 2014) or may provide insight into mutations that potentially affect fitness and therefore persist in future populations (Obermeyer et al. 2022). These models can only provide insight into historical sequences. In contrast, MutaGAN can utilize evolutionary information to make accurate predictions regarding persistence of mutation as well as identify potential new mutations of descendant sequences. Furthermore, MutaGAN predicts future generations at the sequence level, thus providing a mechanism to identify amino acid changes of descendant sequences that can be incorporated into fitness models to inform forecasting of seasonal influenza variants.

### Model reproducibility

Although the mutation profiles are well-reproduced with respect to amino acid and location, MutaGAN’s performance, when evaluated at the single-nucleotide resolution, has a significant room for improvement. The 23.6 per cent and 25.3 per cent capture of standard and sequence true-positive rates and 81.2 per cent and 88.4 per cent prediction of false positives highlight shortcomings of the current trained model. We hypothesized that the model could be improved solely by improving the dataset. There is a high likelihood that the inclusion of swine and avian influenza sequences into the phylogenetic model inhibited the MutaGAN’s ability to fit itself on patterns of HA protein evolution specific to human infection. In addition to a more comprehensive curation of outliers, a larger population of HA protein sequences could be utilized to provide additional diversity to the model. The small number of database records (6,840) used to generate the phylogenetic model was unlikely to capture the full breadth and depth of the true evolutionary landscape of the human H3N2 influenza virus HA protein. By leveraging a larger database of influenza virus surveillance, such as Global Initiative on Sharing All Influenza Data’s EpiFlu (Shu and McCauley 2017), a more complete evolutionary model could be generated and provided to MutaGAN for training, testing, and validation. In addition to the diversity of sequences provided to the phylogenetic tree, constructing this phylogenetic model using time-based Bayesian tree estimation methods could improve the ability of MutaGAN to learn time-related aspects of evolution as well as enable a deeper characterization of the model’s ability to forecast into the future. This approach could also provide us the opportunity to compare our model predictions to those of the experts’ predictions in a given year’s influenza vaccine, as well as look into explainable artificial intelligence techniques to understand why the model made the mutations it did. Future studies will also explore non-deterministic methods of ancestral sequence reconstruction utilizing the simulated nucleotide probabilities per position rather than strictly including the most probable sequence of the internal node for inclusion in the parent–child pair. This would allow us to include in our training data evolutionary pathways that are not part of the ML pathway, but are still probable.

There are also plans to further improve upon the MutaGAN framework’s architecture. There are a number of recent advances in NLP and sequence generation that can be leveraged to further improve this algorithm. These advancements include models like attention (Wu et al. 2016; Vaswani et al. 2017; Zhang et al. 2019), bidirectional encoder representation from transformers (Devlin et al. 2018), and reinforcement learning (Li et al. 2016b; Yu et al. 2017; Keneshloo et al. 2019; Tuan and Lee 2019). These models have shown significant improvement over models relying on LSTMs alone for NLP tasks. When paired with the larger dataset provided by a larger influenza database and non-deterministic methods for ancestral sequence reconstruction, we believe that the fidelity of sequence reconstruction and optimization can be improved. Because operating directly on nucleotide data increases the length of the sequence data from amino acids by a factor of three and further inhibits the recurrent neural network (RNN) identification of long-range structural relationships, it was avoided in this study. However, with the implementation of more robust encoder–decoder architecture, future research could evaluate the feasibility of MutaGAN to operate directly on nucleotide sequences. Doing so would align MutaGAN with the industry standard in phylogenetic analysis and potentially enable improved learning of evolutionary landscapes through the added information of synonymous mutations observed within protein lineages.

## Conclusion

Taken together, we have developed a first-of-its-kind deep learning framework to predict genetic evolution in dynamic biological populations. As a result, we see the potential for this research to play a significant role in public health, particularly in disease mitigation and prevention. With the improvements outlined earlier, if MutaGAN was implemented to simulate how currently circulating pathogens could evolve over time, targeted measures of quarantine and treatment could be more effectively deployed. MutaGAN’s ability to produce full-length protein sequences while simultaneously learning the nuances of evolution lends itself well to the extension to other protein types, creating potential for impact within the domain of multiple diseases.

## Supplementary Material

vead022_Supp

## Data Availability

Influenza virus HA sequences were downloaded from the NCBI IVR. The parent–child pairs used for the training, test, and validation datasets, extracted from the phylogenetic tree, are available at https://github.com/DanBAPL/MutaGAN. Additionally, we have provided the parent–non-child pairs and the bad generated sequences (and models), along with the tokenizer. Code for training the MutaGAN model, performing analysis, and generating figures is available at https://github.com/DanBAPL/MutaGAN.
