# Supplementary material for: MutaGAN: A sequence-to-sequence GAN framework to predict mutations of evolving protein populations
Source: Virus Evol. 2023 Apr 7;9(1):vead022. doi: 10.1093/ve/vead022 (PMC10104372; doi:10.1093/ve/vead022)
Supplement: vead022_Supp [file vead022_supp.zip › Supplemental_table_1.docx]

| Accessions |
| --- |
| 1. LC106066 2. LC106067 3. LC106068 4. LC106069 5. LC106070 6. LC106071 7. LC106072 8. LC106073 9. LC106074 10. LC335983 11. MG912581 12. MG964344 13. MG964360 14. MG964368 15. MG964384 16. MG964408 17. MG964416 18. MG964440 19. MG964448 20. MG964456 21. MG964488 22. MG964512 |
