# Supplementary figures and images for: MutaGAN: A sequence-to-sequence GAN framework to predict mutations of evolving protein populations
Source: Virus Evol. 2023 Apr 7;9(1):vead022. doi: 10.1093/ve/vead022 (PMC10104372; doi:10.1093/ve/vead022)

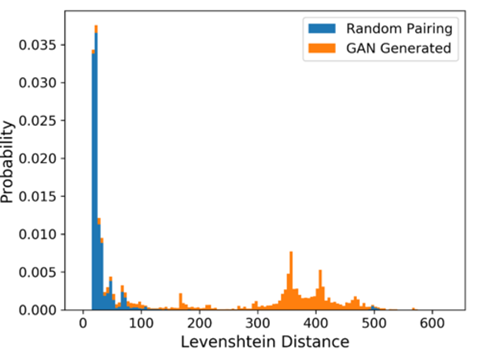

Supplement: vead022_Supp [file vead022_supp.zip › Supplemental_Figure_1.tif]

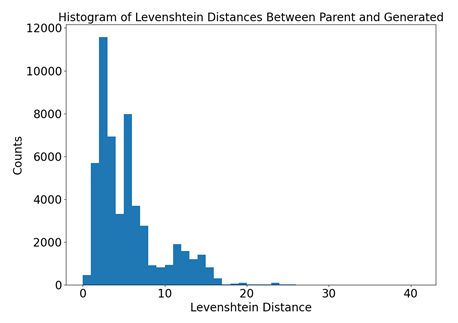

Supplement: vead022_Supp [file vead022_supp.zip › Supplemental_Figure_2.tif]

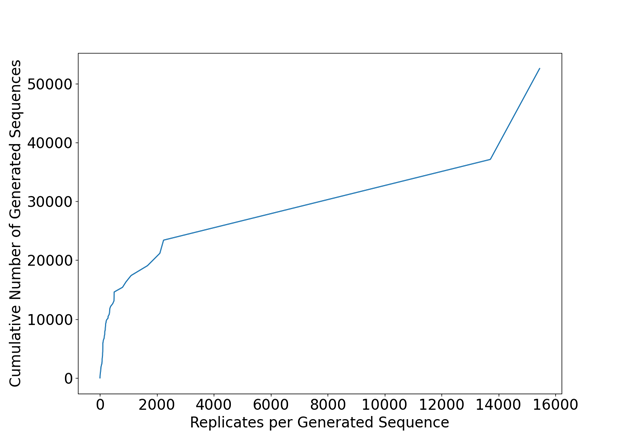

Supplement: vead022_Supp [file vead022_supp.zip › Supplemental_Figure_3.tif]
